# Racial disparities in adequacy of prenatal care during the COVID-19 pandemic in South Carolina, 2018–2021

**DOI:** 10.1186/s12884-023-05983-x

**Published:** 2023-09-23

**Authors:** Emmanuel Fabrice Julceus, Bankole Olatosi, Peiyin Hung, Jiajia Zhang, Xiaoming Li, Jihong Liu

**Affiliations:** 1 Department of Epidemiology and Biostatistics, Arnold School of Public Health, University of South Carolina, Columbia, SC USA; 2 Department of Health Services Policy and Management, Arnold School of Public Health, University of South Carolina, Columbia, SC USA; 3 Department of Health Promotion, Education, and Behavior, Arnold School of Public Health, University of South Carolina, Columbia, SC USA

**Keywords:** Prenatal care, COVID-19, Race, Disparities, South Carolina, Adequacy of prenatal care utilization

## Abstract

**Background:**

During the COVID-19 pandemic, hospitals’ decision of not admitting pregnant women’s partner or support person, and pregnant women’s fear of contracting COVID-19 in hospitals may disrupt prenatal care. We aimed to examine whether prenatal care utilization in South Carolina varied before and during the COVID-19 pandemic, and whether the variation was different by race.

**Methods:**

We utilized 2018–2021 statewide birth certificate data using a pre-post design, including all women who delivered a live birth in South Carolina. The Kotelchuck Index - incorporating the timing of prenatal care initiation and the frequency of gestational age-adjusted visits - was employed to categorize prenatal care into inadequate versus adequate care. Self-reported race includes White, Black, and other race groups. Multiple logistic regression models were used to calculate adjusted odds ratio of inadequate prenatal care and prenatal care initiation after first trimester by maternal race before and during the pandemic.

**Results:**

A total of 118,925 women became pregnant before the pandemic (before March 2020) and 29,237 women during the COVID-19 pandemic (March 2020 – June 2021). Regarding race, 65.2% were White women, 32.0% were Black women and 2.8% were of other races. Lack of adequate prenatal care was more prevalent during the pandemic compared to pre-pandemic (24.1% vs. 21.6%, *p* < 0.001), so was the percentage of initiating prenatal care after the first trimester (27.2% vs. 25.0%, *p* < 0.001). The interaction of race and pandemic period on prenatal care adequacy and initiation was significant. The odds of not receiving adequate prenatal care were higher during the pandemic compared to before for Black women (OR 1.26, 95% CI 1.20–1.33) and White women (OR 1.10, 95% CI 1.06–1.15). The odds of initiating prenatal care after the first trimester were higher during the pandemic for Black women (OR 1.18, 95% CI 1.13–1.24) and White women (OR 1.09, 95% CI 1.04–1.13).

**Conclusions:**

Compared to pre-pandemic, the odds of not receiving adequate prenatal care in South Carolina was increased by 10% for White women and 26% for Black women during the pandemic, highlighting the needs to develop individual tailored interventions to reverse this trend.

**Supplementary Information:**

The online version contains supplementary material available at 10.1186/s12884-023-05983-x.

## Background

The United States (US) lags behind other high-income countries in terms of maternal mortality and infant mortality [1]. With 20.1 maternal deaths per 100,000 live births in 2019, the US ranks last among developed countries [1, 2]. With an infant mortality rate of 5.6 deaths per 1,000 live births in 2019, the US ranks 33rd out of 36 member countries of the Organization for Economic Co-operation and Development (OECD) [1, 3]. Furthermore, racial and ethnic disparities exist in US maternal and infant mortality. Non-Hispanic Black women face 3.2 times higher maternal mortality rate than non-Hispanic White women [2, 4, 5]. Similarly, non-Hispanic Black children experience 2.3 times the infant mortality rate of non-Hispanic Whites [3].

In the joint guidelines for perinatal care, the American College of Obstetricians and Gynecologists and the American Academy of Pediatrics emphasize the importance of adequate prenatal care to achieve optimal maternal and infant outcomes [6]. Prenatal care allows continuously identifying and assessing risks for mothers and offspring, as well as implementing and adapting appropriate care plans. Inadequate prenatal care increases the risk of preterm delivery, low birth weight, neonatal death, and infant death [7–9]. Having four prenatal visits or less, and initiating prenatal care after the first trimester are associated with excess maternal mortality [10]. Black women are less likely to have adequate prenatal care and initiate prenatal care during the first trimester compared to White women [10]. Moreover, Healthy People 2030 has adopted the objective to increase the proportion of pregnant women who receive early and adequate prenatal care to 80.5% [11]. In 2016, 77.1% of women giving birth in the US initiated prenatal care in the first trimester, and 75.6% of women giving birth received adequate prenatal care; in South Carolina, those percentages were respectively 72.0% and 76.0% [12].

During the unprecedented COVID-19 pandemic, many US hospitals made decisions affecting prenatal care experiences. First, several in-person prenatal visits were replaced with virtual visits or phone calls [13–15]. Second, no partner, family member or support person was allowed to attend in-person prenatal visits with the pregnant women [13, 14, 16]. In reaction, pregnant women reported feeling a lack of social support during prenatal visits, including loss of companionship, loss of informational, emotional, and physical help due to the absence of their support persons [14–18]. Pregnant women also reported fear and anxiety about the risk of contracting COVID-19 in hospitals [14, 15, 17]. Worries about safety of in-person prenatal care and about having support persons during prenatal care and delivery were higher for racial and ethnic minorities compared to non-Hispanic White pregnant women [19–22]. In New York State, non-Hispanic Black and Hispanic women more frequently expressed having negative prenatal experiences than non-Hispanic White women during the COVID-19 pandemic [23].

Studies using small samples and/or online convenient samples appear to show a decrease in prenatal visits during the COVID-19 pandemic as a result of worries of pregnant women [15, 16, 18, 21]. Participants reported missing, cancelling, or delaying prenatal visits to decrease the risk of being exposed to COVID-19 [15, 18, 21]. There is a need for population-wide studies assessing the impact of the COVID-19 pandemic on adequacy of prenatal care.

Based on South Carolina statewide birth certificates, this study sought to assess whether the prenatal care utilization varied before and during the COVID-19 pandemic, and whether the variation was moderated by race. We hypothesized that during the pandemic, women were less likely to get adequate prenatal care and initiate prenatal care early than before the pandemic. This study will provide empirical data on how the COVID-19 pandemic may have impacted access to prenatal care, which are much needed for the program development to improve prenatal care utilization during the pandemic and future pandemics.

## Methods

### Design and study population

All pregnant women giving birth in South Carolina from January 2018 to June 2021 were included from a statewide vital records birth certificate dataset. The study was approved by the Institutional Review Boards of the University of South Carolina and the South Carolina Department of Health and Environmental Control, and followed procedures in accordance with the ethical standards of the institutional review boards. Informed consent was waived because this study used de-identified data from a statewide vital records birth certificate dataset and had no more than the minimal risk.

### Outcomes

The primary outcome of interest was the adequacy of prenatal care, which was based on the Kotelchuck Index also known as Adequacy of Prenatal Care Utilization (APNCU). The Kotelchuck Index incorporates the timing of prenatal care initiation and the number of prenatal visits adjusted for gestational age [24] and categorized prenatal care utilization into 4 levels, namely adequate plus, adequate, intermediate, and inadequate. When prenatal care begins by the fourth month of pregnancy, the index is adequate plus if the pregnant women receive ≥ 110% of recommended visits, adequate if she receives 80–109%, and intermediate if she receives 50–79%. If the prenatal care starts after the fourth month of pregnancy, or the pregnant women receives less than 50% of expected visits, the index is inadequate [24]. In this study, prenatal care was considered as adequate if the Kotelchuck Index category was adequate plus or adequate. The secondary outcome was early initiation of prenatal care defined by the prenatal care starting within the first three months of gestation.

### Exposure and covariates

The exposure of interest was pandemic period, which was classified as pre-pandemic if the pregnancy started before March 2020, and pandemic if it started on March 2020 or after. Pregnancy starting time was estimated using the delivery date minus gestational age at the delivery.

Covariates that could potentially affect prenatal care utilization were chosen according to Andersen’s health care utilization model [25]: predisposing factors (race, age, education level), enabling factors (health insurance, participation in supplemental nutrition program as a proxy for income), and need factors (clinical variables of mother and child).

Maternal variables included sociodemographic variables namely race, age, education level, health insurance, participation in supplemental nutrition program. Self-reported race was classified as White, Black, and other. Age was classified as less than 20 years, 20 to 34 years, and 35 years or more, highlighting age groups more at risk of pregnancy complications [26, 27]. Education was categorized into less than high school graduate, high school graduate/associate degree, college graduate or above. Health insurance was classified as private health insurance, Medicaid, or none. Participation in supplemental nutrition program (WIC) was reported as yes or no. Maternal clinical variables such as previous live birth, previous cesarean delivery, diabetes (pre-pregnancy/gestational), hypertension (pre-pregnancy/gestational), and smoking during pregnancy were reported as yes or no. Pre-pregnancy body mass index (BMI) was categorized as underweight or normal weight (< 25.0), overweight (25.0–29.9) and obese (≥ 30.0). Child clinical variables included gestational age and plurality. Gestational age was categorized into preterm (< 37 weeks), and not preterm (≥ 37 weeks). Plurality was reported as single fetus and multiple fetuses.

### Statistical analyses

Counts and percentages were calculated for categorical variables, mean and standard deviation were calculated for numeric variables. For bivariate analyses, Chi square tests were performed to assess the association between the pandemic or covariates and initiation or adequacy of prenatal care. Regarding multivariable analyses, first an unadjusted logistic regression model was performed to test the association between the pandemic and adequacy of prenatal care, and between the pandemic and early initiation of prenatal care. Second, the model was adjusted for the above-mentioned covariates. Finally, the pandemic*race was added to the adjusted model. A separate statistical model was computed for each stratum of race if the interaction term was significant. A p-value < 0.05 was considered statistically significant. SAS 9.4 version (SAS institute, Cary, NC) was utilized for data cleaning and data analysis.

## Results

### Characteristics of the study population

**Fig. 1:**
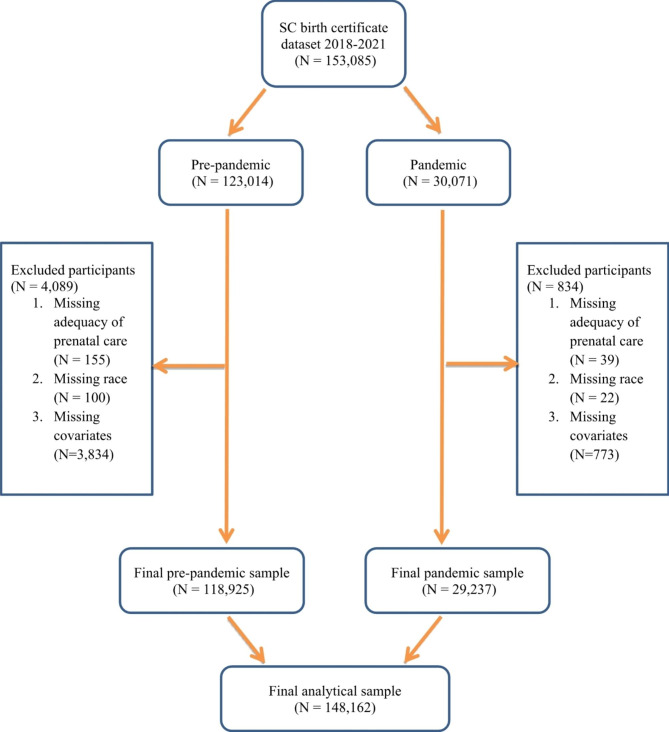
Flowchart of the analytical sample

There were 153,085 women giving birth from January 2018 to June 2021 in South Carolina (Fig. 1). A total of 4,923 observations were excluded due to missing values in adequacy of prenatal care (n = 194), race (n = 122), and covariates (n = 4,607); among covariates, WIC (n = 1,872) and BMI (n = 1,752) contributed most to missing. Those excluded were more likely to be on Medicaid, not participate in WIC, have lower levels of education, have lower pre-pregnancy BMI, delay prenatal care initiation, not receive adequate prenatal care than those remaining (Supplementary Table 1). The remaining 148,162 constituted the analytical sample. Regarding the period, 118,925 women became pregnant during the pre-pandemic period, and 29,237 during the COVID-19 pandemic (Table 1).

**Table 1:** Characteristics of pregnant women giving birth in South Carolina by pandemic and pre-pandemic period, 2018–2021

Characteristics	Total (N = 148,162) N (%)	Pre-Pandemic (N = 118,925) N (%)	Pandemic (N = 29,237) N (%)	P-value^a^
**Age**				**< 0.001**
< 20 years	9162 (6.2)	7494 (6.3)	1668 (5.7)	
20–34 years	116,552 (78.7)	93,494 (78.6)	23,058 (78.9)	
≥ 35 years	22,448 (14.6)	17,937 (15.1)	4511 (15.4)	
**Race**				**0.03**
White	96,653 (65.2)	77,651 (65.3)	19,002 (65.0)	
Black	47,380 (32.0)	37,904 (31.9)	9476 (32.4)	
Other	4129 (2.8)	3370 (2.8)	759 (2.6)	
**Education level**				**0.002**
Less than high school graduate	56,077 (37.9)	45,023 (37.9)	11,054 (37.8)	
High school graduate/ Associate degree	76,799 (51.8)	61,793 (52.0)	15,006 (51.3)	
College graduate or above	15,286 (10.3)	12,109 (10.2)	3177 (10.9)	
**Health Insurance**				**0.02**
Private	63,262 (42.7)	50,837 (42.8)	12,425 (42.5)	
Medicaid	73,560 (49.7)	59,099 (49.7)	14,461 (49.5)	
None	11,340 (7.7)	8989 (7.6)	2351 (8.04)	
**WIC program** ^**b**^				**< 0.001**
Yes	54,372 (36.7)	45,083 (37.9)	9289 (31.8)	
No	93,790 (63.3)	73,842 (62.1)	19,948 (68.2)	
**Previous live birth**				**< 0.001**
Yes	88,502 (59.7)	70,417 (59.2)	18,085 (61.9)	
No	59,660 (40.3)	48,508 (40.8)	11,152 (38.1)	
**Previous cesarean delivery**				0.39
Yes	24,392 (16.5)	19,530 (16.4)	4862 (16.6)	
No	123,770 (83.5)	99,395 (83.6)	24,375 (83.4)	
**Pre-pregnancy or gestational diabetes**				**< 0.001**
Yes	12,290 (8.3)	9587 (8.1)	2703 (9.3)	
No	135,872 (91.7)	109,338 (91.9)	26,534 (90.8)	
**Pre-pregnancy or gestational hypertension**				**< 0.001**
Yes	19,121 (12.9)	14,940 (12.6)	4181 (14.3)	
No	129,041 (87.1)	103,985 (87.4)	25,056 (85.7)	
**Smoking during pregnancy**				**< 0.001**
Yes	10,831 (7.3)	9085 (7.6)	1746 (6.0)	
No	137,331 (92.7)	109,840 (92.4)	27,491 (94.0)	
**Pre-pregnancy BMI** ^**c**^				**< 0.001**
Underweight or normal weight	58,734 (39.6)	47,664 (40.1)	11,070 (37.9)	
Overweight	37,908 (25.6)	30,352 (25.5)	7556 (25.8)	
Obese	51,520 (34.8)	40,909 (34.4)	10,611 (36.3)	
**Gestational age**				**< 0.001**
< 37 weeks	15,987 (10.8)	12,175 (10.2)	3812 (13.0)	
≥ 37 weeks	132,175 (89.2)	106,750 (89.8)	25,425 (87.0)	
**Plurality of fetus**				**0.009**
Single	145,462 (98.2)	116,811 (98.2)	28,651 (98.0)	
Multiple	2700 (1.8)	2114 (1.8)	586 (2.0)	

Percentages may not add up to exactly 100% due to rounding.

^a^P-value derived using chi square with values bolded at < 0.05

^b^Supplemental nutrition program for women, infant and child

^c^Body Mass Index

The racial composition of women who became pregnant during the pandemic (65.0% White women, 32.4% Black women) and before the pandemic (65.3% White women, 31.9% Black women) was similar (p = 0.03) (Table 1). A lower percentage of women participated in WIC during the pandemic compared to before (31.8% vs. 37.9, p < 0.001). During the COVID-19 pandemic a higher percentage of women had diabetes (9.3% vs. 8.1%, p < 0.001) and a higher proportion of births were preterm deliveries (13.0% vs. 10.2%, p < 0.001) compared to before the pandemic. Conversely, a lower percentage of women smoked during pregnancy in the pandemic period compared to before (6.0% vs. 7.6%, p < 0.001).

### Pandemic period and inadequate prenatal care

Overall, 22.1% (32,715) of the women did not receive adequate prenatal care. The percentage of women not receiving adequate prenatal care was higher during the COVID-19 pandemic compared to before (24.1% vs. 21.6%, p < 0.001) (Table 2). The percentage of women not receiving adequate prenatal care was higher among Black women and other race compared to White (respectively 25.9%, 24.3%, and 20.1%, p < 0.001). The odds of lack of adequate prenatal care were higher during the pandemic compared to the pre-pandemic period (adjusted odds ratio (AOR) 1.16, 95% CI 1.12–1.20) (Table 3). Interaction between pandemic and race was significant in both unadjusted and adjusted models (p < 0.001). The odds ratio of pandemic vs. pre-pandemic lack of adequate prenatal care was significant for Black women (AOR 1.26, 95% CI 1.20–1.33) and for White women (AOR 1.10, 95% CI 1.06–1.15), yet not significant for other races.

**Table 2:** Factors associated with adequacy and timing of prenatal care in South Carolina, 2018–2021

Characteristics	Prenatal care Not adequate^a^ N (%)	P-value^b^	Prenatal care Initiated 4–9 months^c^ N (%)	P-value^b^
**Period**		**< 0.001**		**< 0.001**
Pandemic	7043 (24.1)		7940 (27.2)	
Pre- pandemic	25,672 (21.6)		29,753 (25.0)	
**Age**		**< 0.001**		**< 0.001**
< 20 years	3174 (34.6)		3666 (40.0)	
20–34 years	25,270 (21.7)		28,885 (24.8)	
≥ 35 years	4271 (19.0)		5142 (22.9)	
**Race**		**< 0.001**		**< 0.001**
White	19,427 (20.1)		22,277 (23.1)	
Black	12,286 (25.9)		14,202 (30.0)	
Other	1002 (24.3)		1214 (29.4)	
**Education level**		**< 0.001**		**< 0.001**
Less than high school graduate	17,175 (30.6)		19,333 (34.5)	
High school graduate/ Associate degree	13,750 (17.9)		16,343 (21.3)	
College graduate or above	1790 (11.7)		2017 (13.2)	
**Health Insurance**		**< 0.001**		**< 0.001**
Private	8071 (12.8)		9631 (15.2)	
Medicaid	21,314 (29.0)		24,398 (33.2)	
None	3330 (29.4)		3664 (32.3)	
**WIC program** ^**d**^		**< 0.001**		**< 0.001**
Yes	13,172 (24.2)		15,428 (28.4)	
No	19,543 (20.8)		22,265 (23.7)	
**Previous live birth**		**< 0.001**		**< 0.001**
Yes	20,480 (23.1)		23,367 (26.4)	
No	12,235 (20.5)		14,326 (24.0)	
**Previous cesarean delivery**		0.62		0.17
Yes	5357 (22.0)		6121 (25.1)	
No	27,358 (22.1)		31,572 (25.5)	
**Pre-pregnancy or gestational diabetes**		**< 0.001**		**< 0.001**
Yes	1982 (16.1)		2579 (21.0)	
No	30,733 (22.6)		35,114 (25.8)	
**Pre-pregnancy or gestational hypertension**		**< 0.001**		**< 0.001**
Yes	3498 (18.3)		4257 (22.3)	
No	29,217 (22.6)		33,436 (25.9)	
**Smoking during pregnancy**		**< 0.001**		**< 0.001**
Yes	3687 (34.0)		4060 (37.5)	
No	29,028 (21.1)		33,633 (24.5)	
**Pre-pregnancy BMI** ^**e**^		**< 0.001**		**< 0.001**
Underweight or normal weight	13,567 (23.1)		15,329 (26.1)	
Overweight	8368 (22.1)		9654 (25.5)	
Obese	10,780 (20.9)		12,710 (24.7)	
**Plurality of fetus**		**< 0.001**		0.59
Single	32,204 (22.1)		36,994 (25.4)	
Multiple	511 (18.9)		699 (25.9)	

^a^Counts and percentages of adequate prenatal care not shown.

^c^Counts and percentages of prenatal care initiated 1–3 months not shown.

^b^P-value derived using chi square with values bolded at < 0.05

^d^Supplemental nutrition program for women, infant and child

^e^Body Mass Index

**Table 3:** Association between the COVID-19 pandemic and lack of adequate prenatal care in South Carolina, 2018–2021

Race	Period	Prenatal care Not adequate N (%)	Unadjusted Model OR (95% CI)	Adjusted Model^a^ OR (95% CI)
All				
	Pandemic	7043 (24.1)	**1.15 (1.12–1.19)**	**1.16 (1.12–1.20)**
	Pre-pandemic	25,672 (21.6)	1	1
White				
	Pandemic	4021 (21.2)	**1.08 (1.04–1.13)**	**1.10 (1.06–1.15)**
	Pre-pandemic	15,406 (19.8)	1	1
Black				
	Pandemic	2818 (29.7)	**1.27 (1.21–1.34)**	**1.26 (1.20–1.33)**
	Pre-pandemic	9468 (25.0)	1	1
Other				
	Pandemic	204 (26.9)	1.19 (0.99–1.42)	1.19 (0.99–1.43)
	Pre-pandemic	798 (23.7)	1	1

Odds ratio (OR) 95% Confidence interval (95% CI) bolded when significant.

^a^Adjusted for age, education level, health insurance, participation in supplemental nutrition program for women, infant and child, previous live birth, previous cesarean delivery, diabetes, hypertension, smoking during pregnancy, body mass index, and plurality

### Pandemic period and lack of prenatal care and delayed prenatal care initiation

During the pandemic, the percentage of women who did not have prenatal care increased to 3.7% from 1.5% during the pre-pandemic period (p < 0.001). Not having prenatal care varied by race/ethnicity: 1.6% of White women, 2.5% of Black women and 2.5% of other races (p < 0.001). The mean number of prenatal visits was 11.91 during the pandemic compared to 12.24 during the pre-pandemic period.

Overall, 25.4% of the women did not initiate their prenatal care by the third month of pregnancy. The percentage of women not initiating their prenatal care by the third month was higher during the COVID-19 pandemic compared to before (27.2% vs. 25.0%, p < 0.001) (Table 2). Similarly, the percentage of women not initiating their prenatal care by the third month was higher among Black women (30.0%) and other races (29.4%) compared to White women (23.1%), p < 0.001. The odds of delayed prenatal care initiation were higher during the pandemic compared to the pre-pandemic period (AOR 1.12, 95% CI 1.09–1.16) (Table 4). Interaction between pandemic and race was significant in both the unadjusted (p = 0.002) and adjusted (p = 0.02) models. The odds ratio of delayed prenatal care initiation comparing the pandemic to the pre-pandemic period was significant for Black women (AOR 1.18, 95% CI 1.13–1.24) and for White women (AOR 1.09, 95% CI 1.04–1.13), yet not significant for other races.

**Table 4:** Association between the COVID-19 pandemic and delayed prenatal care initiation in South Carolina, 2018–2021

Race	Period	Prenatal care started 4–9 months N (%)	Unadjusted Model OR (95% CI)	Adjusted Model^a^ OR (95% CI)
All				
	Pandemic	7940 (27.2)	**1.12 (1.09–1.15)**	**1.12 (1.09–1.16)**
	Pre-pandemic	29,753 (25.0)	1	1
White				
	Pandemic	4566 (24.04)	**1.07 (1.03–1.11)**	**1.09 (1.04–1.13)**
	Pre-pandemic	17,711 (22.8)	1	1
Black				
	Pandemic	3131 (33.0)	**1.20 (1.14–1.26)**	**1.18 (1.13–1.24)**
	Pre-pandemic	11,071 (29.2)	1	1
Other				
	Pandemic	243 (32.0)	1.16 (0.98–1.38)	1.16 (0.98–1.39)
	Pre-pandemic	971 (28.8)	1	1

Odds ratio (OR); 95% Confidence interval (95% CI) bolded when significant.

^a^Adjusted for age, education level, health insurance, participation in supplemental nutrition program for women, infant and child, previous live birth, previous cesarean delivery, diabetes, hypertension, smoking, body mass index, and plurality

## Discussion

This study shows that the percentage of pregnant women not receiving adequate prenatal care and not initiating prenatal care by the third month of pregnancy in South Carolina increased during the COVID-19 pandemic compared to before the pandemic. The odds of lack of adequate prenatal care and the odds of delayed prenatal care initiation were higher during the pandemic in the models after adjusting for predisposing, enabling and need factors. A difference of 2 − 3% in adequacy of prenatal care and in early prenatal care initiation may seem small, but it is a setback in improving prenatal care adequacy and reaching the Healthy People 2030 target in South Carolina [11]. Online surveys conducted on US women and US obstetric workforce also mentioned a decrease in number of prenatal visits during the COVID-19 pandemic [15, 16, 18, 21]. Knowing the existence of racial disparities in maternal and infant mortality [2–5], and considering that the lack of adequate prenatal care increases the risk of neonatal, infant and maternal death [7–10], racial disparities in access to prenatal care during the COVID-19 pandemic could potentially contribute to the widening of racial gaps.

This study also found that the pre-post variation in adequacy of prenatal care and early prenatal care initiation was moderated by race. For Black women and White women, the odds of lack of adequate prenatal care were higher during the pandemic compared to before. The odds of delayed prenatal care initiation also increased during the pandemic compared to before among Black women and White women. The pre-post increases in lack of adequate prenatal care utilization and delayed prenatal care initiation were more severe in Black women. Surveys and qualitative studies conducted in different US states found higher worries among racial minorities compared to White women regarding contracting COVID-19 during prenatal care and not having support persons [19–22]. In addition, Black women more frequently expressed having negative prenatal experiences than White women during the COVID-19 pandemic [23]. Black women also reported experiencing changes in health care related to discrimination and indicated that systemic racism in the healthcare system had worsened during the pandemic [19].

These findings suggest that racial and ethnic groups face different challenges and barriers to prenatal care utilization particularly during the COVID-19 pandemic. Therefore, policy efforts in maternity health improvements should address the prenatal care utilization by facilitating more patient-centered care to better reach a wide range of pregnant families during the difficult times of the COVID-19 pandemic [28, 29]. In addition, psychosocial support needs to be increased and varied in order to reduce barriers to prenatal care. Findings from this study confirming racial disparities in declining of prenatal care adequacy during the COVID-19 pandemic will help explain the likely mechanism for a possible widening of racial gaps in maternal and perinatal outcomes.

This study is not without limitations. Overall, 3.2% of the observations (2.8% of pandemic and 3.3% of pre-pandemic observations) were excluded because of missing values; and those excluded tend to have lower socioeconomic status and are less likely to receive adequate prenatal care than those included. Therefore, the estimates of inadequate prenatal care or no prenatal care might be underestimated. South Carolina has a small proportion of Hispanics; thus, our results may not be generalizable to Hispanics who are among the groups more affected by the COVID-19 pandemic [30]. There can be discrepancies between birth certificate information and medical records of pregnant women. Furthermore, the birth certificate dataset does not specify whether prenatal visits were in-person or remote, while during the pandemic many in-person visits were changed to remote [13–15]. Our data indicated the differences in participant characteristics such as pre-pregnancy BMI, previous live birth, participation in WIC, gestational age at birth, hypertension, and diabetes, and external factors other than the COVID-19 pandemic, which might also influence the changes in adequacy of prenatal care given the pre-post design. These observed differences were consistent with literature regarding an increase in average BMI and obesity prevalence among US adults during the pandemic [31], as well as an increase in challenges to participation in WIC program [32]. Women with COVID-19 are found to be at higher risk of preterm birth [33]. Nevertheless, our results were adjusted for predisposing (race, age, education level), enabling (health insurance, participation in WIC) and need factors (clinical variables of mother and child) that may affect prenatal care utilization; and the COVID-19 pandemic affected so many external factors (such as employment, childcare, transportation) that the latter are more likely to be mediators than confounders. While the Kotelchuck index measures the timing of prenatal care initiation and the number of prenatal visits, it does not measure the quality of prenatal visits. Despite the limitations, this study has multiple strengths: among them the use of state-wide data, the size of the study population, the use of several measures of prenatal care which assess the different domains (adequacy, timing of initiation, and number of visits) and the consistency of findings regardless of statistical tests, models, and component of prenatal care adequacy.

## Conclusions

In conclusion, during the COVID-19 pandemic pregnant women had higher odds of not receiving adequate prenatal care, and higher odds of delayed prenatal care initiation compared to the pre-pandemic period. Black women were disproportionately affected compared to White women and other races. The decline of prenatal care adequacy and early prenatal care initiation during the COVID-19 pandemic represents a setback in reaching the Healthy People 2030 targets in South Carolina, more severely among Black women. Appropriate and tailored interventions including awareness on prenatal care adequacy, increase in psychosocial support, and changes in prenatal care delivery should be implemented to reverse this trend.

### Electronic supplementary material

Below is the link to the electronic supplementary material.

Supplementary Material 1

## Data Availability

The data that support the findings of this study are available from the South Carolina (SC) Department of Health and Environmental Control (DHEC) and the SC Office of Revenue and Fiscal Affairs, but restrictions apply to the availability of these data, which were used under license for the current study, and so are not publicly available. Data are however available from the authors upon reasonable request and with permission of the South Carolina (SC) Department of Health and Environmental Control (DHEC) and the SC Office of Revenue and Fiscal Affairs. Requests should be directed to the corresponding author Jihong Liu.

## List of abbreviations

APNCU: Adequacy of Prenatal Care Utilization
BMI: Body Mass Index
CI: Confidence Interval
OECD: Organization for Economic Co-operation and Development
OR: Odds Ratio
RR: Rate Ratio
SD: Standard Deviation
US: United States
WIC: Supplemental Nutrition Program for Women, Infants, and Children
