# Supplementary material for: Racial disparities in adequacy of prenatal care during the COVID-19 pandemic in South Carolina, 2018–2021
Source: BMC Pregnancy Childbirth. 2023 Sep 23;23:686. doi: 10.1186/s12884-023-05983-x (PMC10517534; doi:10.1186/s12884-023-05983-x)
Supplement: Supplementary file 1 — Supplementary Material 1 [file 12884_2023_5983_MOESM1_ESM.docx]

| **Supplementary Table 1. Characteristics of pregnant women giving birth** **in South Carolina by inclusion and exclusion status, 2018-2021** | | | |
| --- | --- | --- | --- |
| **Characteristics** | **Included**  (N=148,162)  **N** (%) | **Excluded**  (N= 4,923)  **N (%)** | **P-value*** |
| **Age** |  |  | **<0.001** |
| < 20 years | 9162 (6.2) | 391 (7.9) |  |
| 20-34 years | 116552 (78.7) | 3681 (74.8) |  |
| ≥ 35 years | 22448 (14.6) | 851 (17.3) |  |
| **Race** |  |  | **<0.001** |
| White | 96653 (65.2) | 3142 (65.5) |  |
| Black | 47380 (32.0) | 1425 (29.7) |  |
| Other | 4129 (2.8) | 229 (4.8) |  |
| **Education level** |  |  | **<0.001** |
| Less than high school graduate | 56077 (37.9) | 2102 (47.9) |  |
| High school graduate/ Associate degree | 76799 (51.8) | 1935 (44.1) |  |
| College graduate or above | 15286 (10.3) | 350 (8.0) |  |
| **Health Insurance** |  |  | **<0.001** |
| Private | 63262 (42.7) | 1368 (30.2) |  |
| Medicaid | 73560 (49.7) | 2654 (58.6) |  |
| None | 11340 (7.7) | 505 (11.2) |  |
| **WIC program^a^** |  |  | **<0.001** |
| Yes | 54372 (36.7) | 788 (29.8) |  |
| No | 93790 (63.3) | 1859 (70.2) |  |
| **Previous live birth** |  |  | **<0.001** |
| Yes | 88502 (59.7) | 3099 (63.8) |  |
| No | 59660 (40.3) | 1757 (36.2) |  |
| **Previous cesarean delivery** |  |  | 0.46 |
| Yes | 24392 (16.5) | 791 (16.1) |  |
| No | 123770 (83.5) | 4132 (83.9) |  |
| **Diabetes^b^** |  |  | 0.26 |
| Yes | 12290 (8.3) | 386 (7.8) |  |
| No | 135872 (91.7) | 4537 (92.2) |  |
| **Hypertension^b^** |  |  | 0.44 |
| Yes | 19121 (12.9) | 654 (12.3) |  |
| No | 129041 (87.1) | 4269 (86.7) |  |
| **Smoking^c^** |  |  | **0.003** |
| Yes | 10831 (7.3) | 405 (8.4) |  |
| No | 137331 (92.7) | 4401 (91.6) |  |
| **Pre-pregnancy BMI^d^** |  |  | **<0.001** |
| Underweight or normal weight | 58734 (39.6) | 1299 (43.0) |  |
| Overweight | 37908 (25.6) | 765 (25.4) |  |
| Obese | 51520 (34.8) | 954 (31.6) |  |
| **Gestational age** |  |  | **<0.001** |
| < 37 weeks | 15987 (10.8) | 670 (13.8) |  |
| ≥ 37 weeks | 132175 (89.2) | 4192 (86.2) |  |
| **Plurality of fetus** |  |  | **<0.001** |
| Single | 145462 (98.2) | 4870 (99.0) |  |
| Multiple | 2700 (1.8) | 51 (1.0) |  |
| **Period** |  |  | **<0.001** |
| Pre-pandemic | 118925 (80.3) | 4089 (83.1) |  |
| Pandemic | 29237 (19.7) | 834 (16.9) |  |
| **Adequacy of prenatal care** |  |  | **<0.001** |
| Adequate | 115447 (77.9) | 2977 (63.0) |  |
| Not adequate | 32715 (22.1) | 1752 (37.0) |  |
| **Initiation of prenatal care** |  |  | **<0.001** |
| < 4 months | 110469 (74.6) | 2841 (59.7) |  |
| 4-9 months | 37693 (25.4) | 1915 (40.3) |  |
| Percentages may not add up to exactly 100% due to rounding.  *P-value derived using chi square with values bolded at < 0.05  ^a^Supplemental nutrition program for women, infant and child ^b^pre-pregnancy/gestational  ^c^Smoking during pregnancy ^d^Body Mass Index | | | |
